# A Report on the Progress of Opthalmology and Rhinology

**Published:** 1890-06

**Authors:** R. O. Cotter

**Affiliations:** Macon, Ga.


### A REPORT ON THE PROGRESS OF OPTHALMOLOGY AND RHINOLOGY.—(CONCLUDED.)

BY R. O. COTTER, M. D., MACON, GA.

In my former writings upon hypertrophic catarrh, I am sure that I have not given due prominence to the fact that I consider dyspepsia to play such an important part in the causation and aggravation of these cases. Usually it is in the form of gastro-intestinal catarrh, and before we do anything in the way of surgical treatment, or in connection with the local treatment, we must be sure to do all we can to improve our patient's digestion and assimilation. Of course this means a Herculean task. Diet, exercise, thick-soled shoes to be worn, tobacco smoking to be stopped, and, last and most important of all, coffee drinking must be stopped; and in my opinion tea is nearly as bad. I know nothing better for these dyspeptics than a cup full or so of hot water, sipped one hour before meals; and for the distress which for two or three hours attends the badly digested meal, I know of nothing so good as a cup full of hot water. It will, if anything will, relieve the overburdened stomach by washing the contents onward, and relieves like magic, the distressing feelings of the patient in hypertrophic catarrh. I have many a time made an examination of the patient's nostrils after they had eaten a heavy or indigestible meal, and the vascular turgescence is always greatly aggravated at those times. As to local and surgical means, I attach the very greatest importance to such standard orthodox remedies as chromic acid, the cold wire snare and the saw. We used to waste much chromic acid by applying it upon a flat probe, in the form of a paste. This is the way I now apply it: a small bead of it fused upon this delicate applicator. By the way, I have sometimes seen (not often, however,) even this splendid remedy abused by physicians. They have applied it so vigorously and freely as to have destroyed the normal tissue covering the turbinated bones, and some of these cases thus treated will, in all probability, develop into atropic rhinitis. This is always very unfortunate, and illustrates the fact that these cases require the most discriminating treatment at the hands of a skilled rhinologist, who has made the subject, not the study of a few weeks at some polyclinic, but of years of painstaking observation. Otherwise will not be recognized the wide difference which is demanded in the treatment of a rhinitis caused by a gastro-intestinal catarrh, a mere venous turgescence, a real hyperplasia of tissue, a deflected or hypertrophied septum, an ecchondrosis, or an exostosis of the septum. As I said before, I am not a believer in the galvano-cautery. With it, even skillful hands often do harm by destroying too much tissue or causing adhesive bands, but also, with it "fools rush in where angels fear to tread." I am sure I give you the facts when I assert that the best rhinologists are beginning to leave off its use. I am still a great believer in the use of the Bosworth saw. The little bow saws lately devised by Dr. Roe, of Rochester, are good too. In properly selected cases I use the cold wire snare, especially since I begun to use the Sajous snare. I like the snare better than I used to, and especially is it advisable in the case of patients who live at a distance and are anxious to get home as soon as possible. Of course, in the treatment of hypertrophic catarrh, etc., I attach great importance to the use of properly selected sprays, thrown into the nostrils with the atomizer. I generally employ Dobell's solution, and also a very excellent spray advised by Dr. Carl Seiler, of Philadelphia. It is very similar to Listerine. It is cooling, soothing, and, to some extent, even curative (in acute cases). It is advised that it may be as well and more cheaply prepared from the compressed tablets of its components, but I am quite sure that the solution, as made by Lilly, Rogers & Co., of Baltimore, is far superior to solutions I have seen made from any of the tablets.

It is strange that, at this late day, some physicians who pretend to treat the nose will persist in the use of strong, irritating astringent washes, as sulphate of zinc and copper, chloride of zinc, salicylic acid, etc., etc. I am positive that such things go a long way in the production of these very conditions which we are trying to cure. Douches and irritating washes in the nostrils do positive harm in these cases. Often have patients come to me for whom physicians had prescribed these irritating astringents, and also powders, with their eyes red and streaming, and their nostrils congested, raw and bleeding. That this was all wrong was proven by the fact that proper treatment soon relieved them. Another warning against the improper use of cocaine. Three years ago Dr. W. A. Hammond, of New York, in a paper, extolled the use of a local application of a cocaine solution to relieve the turgescence of the nostrils in hay fever. In a paper upon the subject, soon after, I protested against the continued use of such a powerful stimulant, more especially when placed in the patient's own hands. I now reaffirm more strongly than ever, what I then said. Cocaine should be used as a local anæsthetic in surgical operations, and should never be placed in the patient's hands for him to use. Personally I have never known a case of cocaine habit, and I do not think there is a habitual craving for it—like unfortunates crave whisky or morphine. Of course I do not now refer to those who have engrafted the cocaine on to the morphia habit, but it is known now by careful observers that even weak solutions of cocaine to these hypertrophies in the nostril will really and surely increase this condition. I saw this fact fully illustrated in two of my patients. One of them, a highly intelligent young man, had been long troubled with nasal hypertrophy, and a physician had prescribed a two grain solution of cocaine for him with which to spray the nostrils and relieve the engorgement. It had, on the contrary, greatly intensified it, and so great was his distress at times at the unpleasant congestion of the nostrils and frontal sinuses, that he actually feared that he would lose his reason. Another case: a physician, aged fifty years, had used cocaine tablets in his nostrils to relieve the same condition, with somewhat like effect. The general health of both these patients was undoubtedly impaired by the continuous use of such a powerful stimulant. They both left it off, and I have no reason to doubt their statements that they had no trouble in doing so, as they were positive that they felt no craving for it. I am surprised to see that so high an authority as Dr. F. H. Bosworth, of New York, should recommend, in his new book, the continued use of a spray of cocaine for the relief of nasal hydrorrhoea, a condition which is similar to hypertrophic rhinitis. I will close with some remarks on mouth-breathing.

Continued and careful observation regarding this pernicious habit reveals the fact that it is the cause of, and increases very many of the unpleasant symptoms and conditions above referred to. Usually it is most indulged in at night during sleep. The patient awakes in the morning with a miserably bad taste in his mouth, a dry parched throat and naso-pharynx. It destroys the quality of the voice, brings on intractable sore throats, causes a diseased and hypertrophied condition of the glands of the naso-pharynx, causes the alæ of the nostrils to shrink in, the nostrils to collapse and fill up with adventitious structure, and when the function of the nostril for breathing is thus impaired it gets rapidly worse and worse. Aural catarrh is very frequently brought on from this cause. To relieve these cases we must remove the hypertrophies and other obstructions (in the manner above advised). But if we fail to "keep the track cleared" when we have opened it, the habit of mouth breathing is one of the most obstinate things in the world, and we must remember that the patient indulges in it when he is asleep.

Now there has been various plans, such as straps, harness, etc., to close the mouth, but they have failed. For the past few months, I have been prescribing (in properly selected cases) a little instrument, which is made of celluloid. Where the nostrils have been opened up by proper surgical means, it has proven to be an appliance of more value, as an adjunct, in the treatment of these cases of mouth breathers than anything I have heard of. They have to be fitted, from an impression of the teeth, for each patient. It can be easily and also very comfortably worn between the lips and teeth. It is to be worn when the patient is asleep, and he must, of course, be also impressed with the importance of keeping his mouth closed (that is avoiding mouth breathing) while he is awake. I have prescribed these in about twenty cases, and am highly pleased with the aid they have rendered. This subject of mouth-breathing is a vast one, and it causes a vast deal of trouble of various kinds. If a patient breathes through his mouth we must reflect that, the air which he takes into his bronchial tubes and lungs does not get the benefit of the several degrees of warmth, and the pint of necessary moisture which it should and would get by traversing the natural channel, the nostrils, during twenty-four hours' time. Nor is it filtered of the filthy and poisonous germs which may be afloat in the atmosphere. I hardly think that hypertrophic conditions are so much caused by mouth-breathing as they are made worse by them. I rather think that, aside from deformities, hypertrophies are the result of uncured "colds in the head." However, both hypertrophies, and mouth-breathing are greatly intensified, one by the other.

---

SOAP.—The subject of soap is one of more than ordinary interest, especially to those engaged in the treatment of skin diseases. Mr. B. H. Paul, in an article on Toilet Soap, considered from a chemical point of view (*British Journal of Dermatology*), states that while perfectly neutral soap is at present but rarely obtainable, the production of a really "superfatted" soap, adapted for ordinary use without special disadvantages, is a thing that still remains to be provided in the future. Such a soap would be unquestionably a welcome boon to many, and if the difficulties attending its production can be overcome, it would go far towards preventing the ill effects often experienced as a consequence of using soap that is alkaline. The discomfort of chapped hands and excoriated faces would by that means be at least considerably mitigated. In any case the quality of the soap in regard to neutrality, and the materials from which it has been made, can readily be ascertained. Soap should never be highly perfumed, as the essential oils used for that purpose are liable to have an irritating effect upon the skin. It is still more desirable to avoid using soap of a brilliant color, since the attractive appearance thus communicated to it can only have been the result of adding artificial pigments that may sometimes be very hurtful. All of which we can heartily endorse. Campho-phenique soap, made of the best selected tallow, is at present among the best detergent agents, not only for the healthy skin, but in all those conditions in which there is irritation and in which the use of soap is not contra-indicated.—*St. Louis Med. and Surg. Journal.*

